# Mission of a PAHO/WHO Collaborating Centre and the UN Sustainable Development Goals: achievements and challenges

**DOI:** 10.1590/0034-7167-2024-0140

**Published:** 2025-10-27

**Authors:** Carla Aparecida Arena Ventura, Juliana Gazzotti, Isabel Amélia Costas Mendes, Pedro Fredemir Palha, Maria Helena Palucci Marziale

**Affiliations:** I Universidade de São Paulo. Ribeirão Preto, São Paulo, Brazil

**Keywords:** Sustainable Development, United Nations, Pan American Health Organization, World Health Organization, Nursing Research, Desarrollo Sostenible, Naciones Unidas, Organización Panamericana de la Salud, Organización Mundial de la Salud, Investigación en Enfermería

## Abstract

**Objectives:**

to identify the relationship between activities developed by the Collaborating Centre and the Sustainable Development Goals, during 2010, 2014 and 2018 redesignation processes.

**Methods:**

descriptive and historical-documentary study developed between November and December 2023. Collaborating Centre reports were used as primary sources to collect data, which were analyzed using a qualitative approach. Findings were categorized according to structures identified after the information was organized.

**Results:**

Collaborating Centre work plans in 2010, 2014 and 2018 were mainly related to the Objectives 3 and 4. 2014 Work Plan included Objective 12 and 2018 Work Plan included Objectives 8 and 17.

**Conclusions:**

the Collaborating Centre has successfully fulfilled its mission to advance nursing research development in alignment with targets of the Sustainable Development Goals: knowledge production, capacity building, leadership in knowledge dissemination and transfer of research results to health services with a view to their transformation and to strengthening different nursing networks.

## RESUMEN

**Objetivos:**

identificar la relación entre actividades desarrolladas por el Centro Colaborador y los Objetivos de Desarrollo Sostenible durante los procesos de redesignación 2010, 2014, 2018.

**Métodos:**

estudio descriptivo, histórico-documental, desarrollado entre noviembre y diciembre 2023. Se utilizaron informes del Centro Colaborador como fuentes primarias para recopilación de datos, analizados con enfoque cualitativo. Datos fueron categorizados según estructuras identificadas después de organizar la información.

**Resultados:**

planes de trabajo del Centro Colaborador en 2010, 2014 y 2018 estuvieron relacionados con los Objetivos 3 y 4. El plan de trabajo de 2014 incluyó el Objetivo 12 y de 2018 los Objetivos 8 y 17.

**Conclusiones:**

el Centro Colaborador cumplió su misión de promover el desarrollo de la investigación en enfermería conforme metas de los Objetivos de Desarrollo Sostenible: producción de conocimiento, capacitación, difusión del conocimiento y transferencia de resultados de investigación a servicios de salud, para su transformación, y fortalecimiento de redes de enfermería.

## INTRODUCTION

Collaborating Centres (CCs) are institutions designated by the Director-General of the World Health Organization (WHO) to participate in an international collaborative network that supports WHO programmes at all levels through a range of activities. CCs develop WHO-recommended activities, aligning them with WHO policies and their own internal capacities(1). In January 2000, the WHO Executive Board encouraged Member States to make full use of WHO Collaborating Centres (CCs) as valuable sources of information, services and expertise, while strengthening their national capacity for training, research and collaboration in health development(1).

All WHO collaborating centres in the Region of the Americas are designated as PAHO/WHO collaborating centres, since the Pan American Health Organization functions as the regional office of WHO and in accordance with its constitutional framework. Designation is for an initial period of four years and may be renewed before its expiry(2). As part of the designation/re-designation process, a PAHO/WHO collaborating center establishes terms of reference, which consist of activities agreed with WHO in accordance with its policies. In this context, WHO policies are linked to the goals of the United Nations (UN), including the Sustainable Development Goals (SDGs). At the end of the century, there is a change in the agenda of international cooperation, based on the setting of common goals that direct development funds and strategies in different regions of the world.

Goals have been established, as well as monitoring and evaluation mechanisms to analyze the achievement of the proposed goals (2000-2015). “The United Nations Millennium Declaration, signed in September 2000, commits world leaders to fight poverty, hunger, disease, illiteracy, environmental degradation and discrimination against women”(3). It established the eight Millennium Development Goals (MDGs). As a result, progress toward the MDGs included reducing poverty, improving education and increasing access to safe drinking water. Significant progress was also made on the three health-related goals and targets. Globally, the epidemics of HIV, tuberculosis (TB), and malaria have been substantially reduced, while child and maternal mortality rates have declined significantly (by 53% and 44%, respectively, since 1990), although the MDG targets have not been fully met(4).

The MDGs have had a significant impact on the global development debate and have led to substantial increases in development assistance. However, several limitations have emerged. These include a narrow focus that has led to a fragmented approach to health and disease programs, insufficient attention to strengthening health systems, reliance on a “one-size-fits-all” model for development planning, and an emphasis on aggregate targets rather than equitable outcomes(5).

In light of this, at the Rio+20 Conference (the United Nations Conference on Sustainable Development) in Rio de Janeiro in June 2012, the international community launched a process to create a new set of SDGs, with the aim of maintaining the momentum created by the MDGs and establishing a global development framework beyond 2015. In July 2014, the UN General Assembly’s Open Working Group (OWG) proposed a document of 17 goals for consideration and adoption by the General Assembly, which took place in September 2015. This document laid the foundation for the new SDGs and the global development agenda for the period 2015 to 2030(4).

The 2030 Sustainable Development Agenda builds on the MDGs. While poverty eradication, health, education, and food security and nutrition remain key priorities, the SDGs include a wide range of economic, social, and environmental goals(4).

The 17 goals and 169 targets of the new development agenda, including a specific goal on health with 13 targets, integrate the dimensions of sustainable development, focusing on people, planet, prosperity, peace and partnership. The health goal is broad: “Ensure healthy lives and promote well-being for all at all ages”(4). Health has a critical role to play, both as a major contributor to and beneficiary of sustainable development policies. There are numerous linkages between the health goal and other goals and targets, highlighting the integrated approach that underpins the SDGs. Universal health coverage (UHC), one of the 13 targets within the health goal, serves as a comprehensive framework for implementing a broad and ambitious health agenda in all countries. Therefore, in addition to SDG number 3, other targets contribute to the promotion of health, recognizing its social, environmental, and economic significance, along with a multidisciplinary approach that includes targets such as eradicating poverty, ensuring quality education, achieving gender equality, and providing clean water and sanitation(4).

In this context, CCs play a fundamental role in the decentralized process of achieving the SDGs by helping to train human resources, adapt solutions to local realities, form partnerships and networks that connect different sectors of society in search of innovative approaches, and disseminate good practices(1). In Brazil, the Ribeirão Preto School of Nursing of the University of São Paulo, a university center located in the interior of the state of São Paulo, played an innovative and leading role with the designation of a CC in the 1980s, the only one in the field of nursing in the country, and it has maintained successive redesignations since then. In addition, it collaborates with PAHO Brazil and the Ministry of Health to support the implementation of public policies.

## OBJECTIVES

To identify the relationship between the activities developed by the PAHO/WHOCC for Nursing Research Development, located at the University of São Paulo, Ribeirão Preto College of Nursing (EERP-USP), and the UN Sustainable Development Goals (SDGs) during the 2010, 2014 and 2018 redesignation processes.

## METHODS

### Ethical aspects

The sources used in this study are publicly available and therefore did not require review by a research ethics committee.

### Design, place and period

This is a qualitative and descriptive study, of historical-documentary nature, developed between November and December 2023 at EERP-USP. Data were collected from nine annual reports of the PAHO/WHOCC for Nursing Research Development sent to the WHO. Annual reports are automatically requested by WHO via email and refer to the last twelve months of their previous designation. For this study, nine annual reports (from 2010 to 2018) were used for data collection.

### Population and sample

Primary sources were used, especially the processes and reports produced by the EERP-USP in 2010, 2014, and 2018. It is worth noting that EERP-USP was first designated as a World Health Organization (WHO) Collaborating Center for Nursing Research Development in 1988. Subsequently, the EERP-USP was redesignated in 1992, 1997, 2002, 2006, 2010, 2014 and 2018 for a period of 4 years in each redesignation and has been working in line with the SDGs since then. The last redesignation was in 2022 and will last until 2026. This last redesignation was not included because some of the Terms of Reference (TORs) were partially modified.

### Study protocol

A work plan is developed that includes identifying and locating sources, analyzing primary source scripts, and connecting the activities developed to the SDGs.

### Analysis of the results

Descriptive analyses of the activities included in the ToRs, which are reported annually to WHO, were conducted. This was used to determine whether the activities carried out by the CCs addressed the SDG targets. Two researchers conducted the analysis independently, and in case of divergence in the relationship between activities and targets, a third researcher was consulted to help reach a consensus.

The results were classified according to the relevant structures identified after organizing the information. In 2010, the CC had 3 ToRs and 11 activities (2 related to ToR1, 7 related to ToR2 and 2 related to ToR3) and in 2014, the CC had 3 ToRs and 9 activities (3 related to ToR1, 4 related to ToR2 and 2 related to ToR3). In 2018, the CC had 3 ToRs and 10 activities (3 related to ToR1, 4 related to ToR2 and 3 related to ToR3).

## RESULTS

Terms of Reference (redefined in 2010, 2014, and 2018):

TR#1 Collaborate with PAHO/WHO in strengthening nursing research for the development of nursing human resources and nursing practice in priority areas.

TR#2 Collaborate with PAHO/WHO to strengthen and expand the dissemination of health information and knowledge with a focus on nursing and health.

TR#3 Contribute with PAHO/WHO to strengthening nursing faculty development through research training and updating to improve scientific quality and nursing curricula.

The results are summarized in Chart 1.

**Chart 1:** Objectives, Goals, and Activities developed by the Collaborating Center

GOALS*	TARGETS*	CC ACTIVITIES PERFORMED
Goal 3. Ensure healthy lives and promote well-being for all at all ages	3.1 Reduce the global maternal mortality ratio to less than 70 per 100,000 live births by 2030.	The CC developed strategies related to the Safe Motherhood Initiative in Latin America and the Caribbean. As partners in this initiative, two CC members who are experts in women’s health participated in the Global Alliance for Nursing and Midwifery – GANM/PAHO for the Development of Leadership in Midwifery, in Task Forces to Achieve Safe Motherhood, taking the lead/facilitator position of Community Practice Nursing and Midwifery for Making Pregnancy Safer, especially in Portuguese and Spanish speaking countries.
Goal 3. Ensure healthy lives and promote well-being for all at all ages	3.1 Reduce the global maternal mortality ratio to less than 70 per 100,000 live births by 2030.	The CC participated in the development of a collaborative research project in partnership with the University of Alberta. The project, entitled “Immigrant and Aboriginal Women’s Food Choice and Practice”, examined how the beliefs and health practices of immigrant and Aboriginal women during pregnancy influence their food choices and how these choices, in turn, affect their overall health.
Goal 3. Ensure healthy lives and promote well-being for all at all ages	3.2 End, by 2030, all preventable deaths of newborns and children under five, with all countries aiming to reduce neonatal mortality to no more than 12 per 1,000 live births and under-five mortality to no more than 25 per 1,000 live births.	The CC participated in the development of a multicenter research project to teach nursing schools in Latin America and the Caribbean how to prevent childhood diseases.
Goal 3. Ensure healthy lives and promote well-being for all at all ages	3.2 End, by 2030, all preventable deaths of newborns and children under five, with all countries aiming to reduce neonatal mortality to no more than 12 per 1,000 live births and under-five mortality to no more than 25 per 1,000 live births.	The CC participated in the development of a multicenter research project funded by the Brazilian Federal and State Research Agencies – Bill & Melinda Gates Foundation – Fogarty International Center, which aimed to implement and evaluate the effectiveness of the innovative intervention Child Friendly Hospital Initiative for Neonatal Units.
Goal 3. Ensure healthy lives and promote well-being for all at all ages	3.2 End, by 2030, all preventable deaths of newborns and children under five, with all countries aiming to reduce neonatal mortality to no more than 12 per 1,000 live births and under-five mortality to no more than 25 per 1,000 live births.	The CC participated in the development of a joint research project with the Florence Nightingale School of Nursing and Midwifery-King’s College London on essential needs and vulnerabilities in child care in the context of primary health care. A CC member was one of the coordinators of this project, which resulted in a published article entitled “Child safety from the perspective of essential needs”.
Goal 3. Ensure healthy lives and promote well-being for all at all ages	3.2 End, by 2030, all preventable deaths of newborns and children under five, with all countries aiming to reduce neonatal mortality to no more than 12 per 1,000 live births and under-five mortality to no more than 25 per 1,000 live births.	The CC participated in a collaborative research project aimed at improving a digital learning object (e-baby) to include an emotional design to evaluate the impact of its use on the emotional responses of Brazilian and Portuguese nursing students. The project was developed in partnership with the School of Nursing of Coimbra, Portugal.
Goal 3. Ensure healthy lives and promote well-being for all at all ages	3.2 End, by 2030, all preventable deaths of newborns and children under five, with all countries aiming to reduce neonatal mortality to no more than 12 per 1,000 live births and under-five mortality to no more than 25 per 1,000 live births.	This CC, in partnership with the Federal University of Bahia, the Indian Institute of Public Health, and the London School of Hygiene and Tropical Medicine, developed a multi-center research project entitled “Effects of congenital neurological manifestations associated with Zika virus on child cognitive development: a prospective cohort study in the context of basic health care” to assess the spectrum of neurocognitive outcomes up to the first three and a half years and to determine performance differences between children exposed and not exposed to the neurological consequences of maternal infection with Zika virus during pregnancy.
Goal 3. Ensure healthy lives and promote well-being for all at all ages	3.2 End, by 2030, all preventable deaths of newborns and children under five, with all countries aiming to reduce neonatal mortality to no more than 12 per 1,000 live births and under-five mortality to no more than 25 per 1,000 live births.	The CC developed a multicenter research in partnership with the Ribeirão Preto School of Economics, Business Administration and Accounting of the University of São Paulo (FEARP-USP) and the Department of Child, Youth and Family Studies, College of Education and Human Sciences of the University of Nebraska-Lincoln, USA, entitled “Choosing the type of care for young child: child development and family needs”. This study looked at how families plan for the care of their future baby, how these plans change after the baby is born, and how well the child’s care services meet the needs of the child and the family.
Goal 3. Ensure healthy lives and promote well-being for all at all ages	3.2 End, by 2030, all preventable deaths of newborns and children under five, with all countries aiming to reduce neonatal mortality to no more than 12 per 1,000 live births and under-five mortality to no more than 25 per 1,000 live births.	The CC developed collaborative research with Western Sydney University, Australia. The aim was to evaluate the effectiveness of atrial acupuncture in lactating women to improve breastfeeding conditions and increase daily milk production.
Goal 3. Ensure healthy lives and promote well-being for all at all ages	3.3 By 2030, end the epidemics of AIDS, tuberculosis, malaria and neglected tropical diseases, and combat hepatitis, water-borne diseases and other communicable diseases.	The CC integrated international and national tuberculosis and TB/HIV networks and coordinated research projects in Brazil (Northeast, South and Southeast, and research in Africa) on the evaluation of primary care in tuberculosis treatment, health services management and nursing interventions for tuberculosis control in primary care, and policy transfer for tuberculosis control.
Goal 3. Ensure healthy lives and promote well-being for all at all ages	3.5 Strengthen prevention and treatment of substance abuse, including drug abuse and harmful use of alcohol.	The CC participated in the development of a multicenter research project aimed at studying the consequences of drug use among students in nine universities in six Latin American countries and three Caribbean countries. The universities involved were the University of São Paulo at Ribeirão Preto College of Nursing, Universidad de Concepción, Universidad de Costa Rica, Universidad Evangélica de El Salvador, Northern Caribbean University, Benemérita Universidad Autónoma de Puebla, Universidad Nacional Autónoma de Nicarágua, Anton de Kon University of Suriname, and University of the Southern Caribbean. The study used the same 70-item research instrument modified with questions on sociodemographic characteristics and knowledge of the effects of alcohol, marijuana and cocaine on academic performance.
Goal 3. Ensure healthy lives and promote well-being for all at all ages	3.5 Strengthen prevention and treatment of substance abuse, including drug abuse and harmful use of alcohol.	The CC is part of the organizing committee of the biennial Meeting on Mental Health Researchers and Psychiatric Nursing Specialists. The aim of these meetings is to reflect on where the field of mental health is heading in terms of care, teaching and research, through the presentation of studies by professionals and academics in the field.
Goal 3. Ensure healthy lives and promote well-being for all at all ages	3.8 Achieve universal health coverage, including financial protection, access to quality essential health services, and access to safe, effective, quality and affordable essential medicines and vaccines for all.	One of the necessary changes in nursing education is to increase the number of nurses with doctoral degrees in Latin America and the Caribbean. This will help develop professionals who are equipped to lead the scientific advancement of the nursing field. In December 0f 2016, in partnership with PAHO, members of this CC were among those responsible for reviewing and making proposals in the document “Action Plan for Doctoral Programs in Latin America and the Caribbean”. The objective of the document was to propose strategies to promote the training of doctoral students in the Latin American and Caribbean region through technical, academic and research cooperation, and the development and consolidation of graduate programs that support the generation and transfer of knowledge and technological innovation to contribute to the transformation of the health reality of the countries. In addition, in 2017, the document “Doctoral Education in Nursing in Latin America and the Caribbean” was published. It provides an analysis of doctoral programs in Latin America and the Caribbean, accompanied by an action plan.
Goal 3. Ensure healthy lives and promote well-being for all at all ages	3.8 Achieve universal health coverage, including financial protection, access to quality essential health services and access for all to safe, effective, quality and affordable essential medicines and vaccines.	The CC was responsible for developing a compendium of exemplary Brazilian case studies that showcase best practices in care to promote universal access to health and universal health coverage. Members of this CC develop research projects aimed at achieving universal access to health in the Americas, the results of which are published on the CC website.
Goal 3. Ensure healthy lives and promote well-being for all at all ages	3.C Substantially increase health financing and the recruitment, development, training and retention of health workers in developing countries, particularly in the least developed countries and small island developing states.	The CC participated in the Brazil-Uruguay International Health Cooperation Program, supported by the Brazilian Ministry of Health and PAHO. Research projects were developed, such as the “Project on International Cooperation in Health: Nursing Technology Transfer in Census Research”to support the exchange of experiences, knowledge and technologies related to the structuring and development of a census study on the nursing workforce in Uruguay; “Sizing the workforce: classification of practices in primary health care”; “Method of sizing the workforce in primary health care”; and “Sizing of personal and characterization of skills of primary health care for collaborative practice”.
Goal 3. Ensure healthy lives and promote well-being for all at all ages	3.C Substantially increase health financing and the recruitment, development, training and retention of health workers in developing countries, particularly in the least developed countries and small island developing states.	The CC developed a research project entitled “Animated Infographic on Interventions during Labor”. The objectives were to review the scientific literature on recommended obstetric interventions, the procedures for which benefits and harms should be assessed, and the procedures performed during the stages of labor; to develop an animated infographic on interventions during labor; to review and validate the developed animated infographic; and to evaluate the ability of the infographic to stimulate learning among health professionals related to interventions during labor.
Goal 3. Ensure healthy lives and promote well-being for all at all ages	3.C Substantially increase health financing and the recruitment, development, training and retention of health workers in developing countries, particularly in the least developed countries and small island developing states.	The CC developed a research project entitled “Virtual laboratory for skills development in entrepreneurship and innovation of high social impact”, which aimed to develop and evaluate the quality and usability of a game (prototype) focused on entrepreneurship and innovation of high social impact. Its objective was to promote the dissemination of knowledge and strengthen a culture of innovation and entrepreneurship, encouraging the incorporation and application of these principles in the identification of problems and solutions for health care, education and health and care management. Through online activities and the availability of informative and educational digital materials, the game served as a virtual space for simulation and experimentation, transforming innovative ideas into new processes, products, services and high social impact enterprises.
Goal 3. Ensure healthy lives and promote well-being for all at all ages	3.C Substantially increase health financing and the recruitment, development, training and retention of health workers in developing countries, particularly in the least developed countries and small island developing states.	The CC also developed a research project on interprofessional collaborative practice in nursing education in Brazil to analyze the education of nurses regarding interprofessional collaborative practice; to analyze the politicalpedagogical projects and curricula of nursing courses in Brazil, identifying the development of interprofessional actions; and to identify aspects that facilitate and hinder interprofessional education of nurses.
Goal 3. Ensure healthy lives and promote well-being for all at all ages	3.D Strengthen the capacity of all countries, particularly developing countries, for early warning, risk reduction and management of national and global health risks.	The CC participated in a multicenter research project in partnership with the University of Alabama at Birmingham School of Nursing, USA and the Universidad Nacional Autónoma de México, Mexico to identify nursing competencies in global health. The objective was to validate the Global Health Competency Survey questionnaire for the Brazilian culture and to determine the level of agreement among Brazilian nursing professors regarding the global health competencies that students should acquire during their undergraduate nursing education.
Goal 4. Ensure inclusive and equitable quality education and promote lifelong learning opportunities for all	4.3 By 2030, ensure equal access for all women and men to affordable and quality technical, vocational and tertiary education, including university education.	The CC collaborates with PAHO in the organization and maintenance of the Pan American Directory of Schools of Nursing. An extensive electronic search was carried out to feed the database, which aims to collect information from nursing institutions throughout the Americas and make it available to the general public in order to increase the visibility of nursing schools and provide a range of options of recognized schools that offer accessible and quality education.
Goal 4. Ensure inclusive and equitable quality education and promote lifelong learning opportunities for all	4.4 Substantially increase by 2030 the number of youth and adults with relevant skills, including technical and vocational skills, for employment, decent work and entrepreneurship.	The CC participated in a collaborative research project focused on simulation as a teaching strategy for nursing education. This project was developed in partnership with the School of Nursing of Coimbra and analyzed scientific evidence related to simulation in nursing education in order to validate pedagogical materials according to cultural, social and pedagogical aspects, to improve clinical experiences based on the development of clinical competencies, relationship, ethics and critical thinking, to analyze the implementation process of simulated learning experiences from the perspective of professors and students, and to analyze what they have learned during these simulated experiences.
Goal 4. Ensure inclusive and equitable quality education and promote lifelong learning opportunities for all	4.4 Substantially increase by 2030 the number of youth and adults with relevant skills, including technical and vocational skills, for employment, decent work and entrepreneurship.	CC partnered with the Ministry of Health of Brazil to train health workers in Haiti.
Goal 4. Ensure inclusive and equitable quality education and promote lifelong learning opportunities for all	4.4 Substantially increase by 2030 the number of youth and adults with relevant skills, including technical and vocational skills, for employment, decent work and entrepreneurship.	To strengthen primary care, this CC developed a continuing education project in health. The project was carried out in twenty-four cities and involved more than one hundred workers from different backgrounds, with the participation of researchers and managers from different institutions. Among the activities, the participation of researchers in a course organized by Brazilian and North American institutions should be highlighted, which promoted research and training in the field of labor economics and health education for researchers from Mercosur and the Andean region.
Goal 4. Ensure inclusive and equitable quality education and promote lifelong learning opportunities for all	4.7 By 2030, ensure that all learners acquire the knowledge and skills necessary to promote sustainable development, including through education for sustainable development and sustainable lifestyles, human rights, gender equality, the promotion of a culture of peace and non-violence, global citizenship and the appreciation of cultural diversity and the contribution of culture to sustainable development.	The CC participated in a collaborative project in partnership with the University of Surrey. The aim was to compare legislation from Brazil and the UK on the human rights of patients during involuntary admission to psychiatric hospitals, based on the WHO Checklist on Mental Health Legislation.
Goal 4. Ensure inclusive and equitable quality education and promote lifelong learning opportunities for all	4.7 By 2030, ensure that all learners acquire the knowledge and skills necessary to promote sustainable development, including through education for sustainable development and sustainable lifestyles, human rights, gender equality, the promotion of a culture of peace and non-violence, global citizenship and the appreciation of cultural diversity and the contribution of culture to sustainable development.	The CC developed a collaborative research project in partnership with the Faculty of Nursing at the University of Alberta and the John Dossetor Health Ethics Centre in Edmonton, Canada. The objectives were to explore the relationship between mental health, human rights and ethics; to understand the mechanisms and procedures for the protection of persons with mental disorders admitted to psychiatric hospitals in Canada; to compare the mechanisms and procedures for the protection of the human rights of persons with mental disorders in Canada with those in Brazil; and to develop a training program focused on human rights and ethics in mental health care for health professionals.
Goal 4. Ensure inclusive and equitable quality education and promote lifelong learning opportunities for all	4.B By 2020, substantially increase globally the number of scholarships available to developing countries, in particular least developed countries, small island developing States and African countries, for enrolment in higher education, including vocational education and training and information and communication technology, technical, engineering and scientific programmes, in developed countries and other developing countries.	The CC received nurses from Mozambique through the “*Mujeres por Africa*” program. This program was formalized through an agreement between Brazil, Spain and Africa. The nurses were enrolled in a professional training and continuing education and developed activities of clinical practice and theoretical learning in different areas such as women’s health, comprehensive health care for children and adolescents, evaluation of medical tests, wound care, breast cancer and orthopedics, among others. The program contributed to the development of human resources in Mozambique by enhancing the professional skills and competencies of these nurses.
Goal 4. Ensure inclusive and equitable quality education and promote lifelong learning opportunities for all	4.B By 2020, substantially increase globally the number of scholarships available to developing countries, in particular least developed countries, small island developing States and African countries, for enrolment in higher education, including vocational education and training and information and communication technology, technical, engineering and scientific programmes, in developed countries and other developing countries. Each year, the CC hosted a delegation of nursing students from Agostinho Neto University, Angola, for a three-month clinical internship.	The students were funded through this partnership and participated in courses in the following areas: maternal and infant care, diseases, drugs and alcohol, targeting the country’s most pressing health issues.
Goal 4. Ensure inclusive and equitable quality education and promote lifelong learning opportunities for all	4.B By 2020, substantially increase globally the number of scholarships available to developing countries, in particular least developed countries, small island developing States and African countries, for enrolment in higher education, including vocational education and training and information and communication technology, technical, engineering and scientific programmes, in developed countries and other developing countries.	The CC also had a partnership with Agostinho Neto University through the implementation of a Master’s in Nursing and Midwifery in the country. As part of this program, members of this CC offered face-to-face courses and regularly visit Agostinho Neto University as part of their leadership role in the program.
Goal 4. Ensure inclusive and equitable quality education and promote lifelong learning opportunities for all	4.C Substantially increase, by 2030, the supply of qualified teachers, including through international cooperation for teacher training in developing countries, in particular least developed countries and small island developing States.	The CC participated in the development of a multicenter research project in partnership with the Université de Cergy Pontoise, France, to examine health promotion and disease prevention in professional nursing education in both countries.
Goal 4. Ensure inclusive and equitable quality education and promote lifelong learning opportunities for all	4.C Substantially increase, by 2030, the supply of qualified teachers, including through international cooperation for teacher training in developing countries, in particular least developed countries and small island developing States.	The CC has created a virtual course entitled “Scientific Methodology and Strategies for Dissemination of Knowledge”, offered in Portuguese, Spanish and English. This course aims to improve the understanding of research development in health and nursing. It is an open online course, with the Spanish and English versions available on the Virtual Campus for Public Health (VCPH/PAHO).
Goal 8. Promote sustained, inclusive and sustainable economic growth, full and productive employment and decent work for all	8.5 Achieve, by 2030, full and productive employment and decent work for all women and men, including young people and persons with disabilities, and equal pay for work of equal value.	The CC offers postgraduate courses and develops studies in the field of occupational health, training masters and doctoral students to carry out actions and contribute to the promotion of healthy work environments through the identification and intervention in occupational risk situations. With the advent of the covid-19 pandemic, the CC participated in 2021 in a research project in partnership with PAHO/WHO entitled “Impact of covid-19 on health workers in Brazil: working conditions and physical and mental illness”, whose objective was to analyze the impact of covid-19 on working conditions and covid-19 infection among health workers in Brazil during the pandemic, based on epidemiological data and scientific evidence. The data collected were sent to the WHO and included in its international indicator reports. In Brazil, it was also presented to managers and policy makers during the “International Webinar: Impacts of covid-19 on the working conditions and health of health professionals in Brazil” promoted by this CC in partnership with the Ministry of Health and PAHO/Brazil.
Goal 12. Ensure sustainable consumption and production patterns	12.4 Achieve, by 2020, environmentally sound management of chemicals and all wastes throughout their life cycle, in accordance with agreed international frameworks, and significantly reduce their release to air, water and land in order to minimize their adverse effects on human health and the environment.	The CC participated in a collaborative research project on Environmental Management Tool for Risk Assessment in Watersheds. The project received funding from the Spanish Funding Agency – Programa de Cooperación Interuniversitaria e Investigación Científica (PCI) and was developed in partnership with the Universitat Rovira i Virgili, Spain.
Goal 17. Strengthen the means of implementation and revitalize the global partnership for sustainable development	17.9 Strengthen international support for effective and targeted capacity-building in developing countries to support national plans for the implementation of all the Sustainable Development Goals, including through North-South, South-South and triangular cooperation.	The CC was contacted by the Regional Advisor on Nursing and Allied Health Personnel/Health Systems and Services of the Pan American Health Organization (PAHO/WHO) to collaborate with the Ministry of Public Health of Guyana to build human resource capacity for nursing and midwifery in Guyana to move nursing education and practice to the master’s and doctoral levels. A work plan and agreement were developed and approved by both institutions. The call for the selection process was launched in April 2018, and the director of this CC traveled to Guyana to serve on the committee responsible for selecting 13 students, eight for the Master’s program and five for the PhD program. The students began their graduate programs in September 2018. This activity is integrated into the overarching goal of promoting the development and strengthening of nursing research in Latin America and the Caribbean through human resource development and education.

*PS: The goals and targets of the SDGs are written exactly according to the reference 4.

## DISCUSSION

The World Health Organization’s (WHO) Collaborating Centers (CCs) are critical to advancing global health initiatives. These centers, which include research institutions, universities and other designated entities, are tasked with specific activities that support WHO’s strategic programmes. Their contributions are critical to the effective implementation of global health goals, such as the UN Sustainable Development Goals, particularly SDG 3, which aims to ensure healthy lives and promote wellbeing for all at all ages. Their main functions include conducting research that addresses priority health issues, developing and disseminating knowledge, training human resources, and implementing global agendas and global health emergencies(1). In light of this, the 2010, 2014 and 2018 CCs Work Plans were mainly related to SDGs 3 and 4 “Ensure healthy lives and promote well-being for all at all ages”(4) and “Ensure inclusive and quality education for all and promote lifelong learning”(4). The 2014 work plan also includes SDG 12, “Ensure sustainable consumption and production patterns”(4) and the 2018 work plan also includes SDG 8, “Promote sustained, inclusive and sustainable economic growth, full and productive employment and decent work for all”(4) and SDG 17, “Strengthen the means of implementation and revitalize the global partnership for sustainable development”(4). In relation to SDG 8, it is highlighted that this CC is concerned with the capacity of human resources to develop policies and contribute to the promotion of healthy work environments through the identification and intervention in situations of occupational risk. In addition, during the covid-19 pandemic, the CC, in collaboration with PAHO/WHO and the Brazilian Ministry of Health, identified indicators of risk of SARS-Cov-2 infection, illness and death due to covid-19 among health care workers.

In terms of targets, in 2010 the activities related to targets 3.1 (2), 3.2 (4), 3.3, 3.5, 3.C, 4.3, 4.4, 4.7 and 4.B (3). In 2014, they related to objectives 3.2 (3), 3.5, 3.8, 3.D, 4.4 (2), 4.C and 12.4. In 2018, they related to 3.8, 3.C (3), 4.7, 4.C and 17.9.

CC activities related to the SDGs were grouped into four categories:

1. research activities focusing on maternal health, child health, communicable diseases, prevention and treatment, and mental health;
2. strengthening nursing research through human resource development and educational advancement;
3. disseminating nursing knowledge; and
4. strengthening nursing networks.

Among the goals of the SDGs, those related to health are fundamental to global development, especially for the most vulnerable populations. According to Buss, there is a consensus among authors that health, “understood as a healthy population and not simply the absence of disease, is fundamental to economic and social development”(6). At the same time, SDG 3, “Ensure healthy lives and promote well-being for all at all ages”, “will be influenced by measuring the outcomes of many other economic, social, and environmental SDGs”(7).

As can be seen in the grouping of activities developed by this CC, most of them are related to SDG 3, demonstrating the focus on prevention and integrated and patient-centered care. The research projects carried out by this CC are important tools to contribute to the reduction, control and treatment of diseases, and are therefore in line with what has been established by the SDGs and in favor of achieving their goals(3). This work will also be developed in convergence with the PAHO/WHO objectives(2). According to PAHO, this convergence strengthens the strategic function of the Collaborating Centers, particularly for the achievement of SDG 3.“Given their global reach and the focus that many of them place on capacity building at the local, regional, and global levels, these Centers provide key tools for achieving SDG 3 and other future goals”(8). These activities are included in the annual reports submitted to WHO.

PAHO/WHO is committed to the implementation of the 2030 Agenda for the SDGs. An important document for this is the 13th General Plan of Work of the World Health Organization, which was approved at the 71st World Health Assembly in May 2018. The document aims to create the conditions for improving the health of populations in the next five years after its establishment. WHO acknowledges that its success depends on aligning its efforts with the SDGs. “GPW 13 is based on the SDGs and is relevant to all countries – low, middle and high income. Health is fundamental to the SDGs, and in an interconnected world, WHO’s role in providing global public goods that help ensure the health of all people within and across national borders has never been more important. WHO’s unique status as a scienceand evidence-based organization that sets globally applicable norms and standards makes it indispensable in a rapidly changing world. The Organization’s strong voice for health and human rights is essential to ensure that no one is left behind. Broad and sustained efforts are needed to build a community that works for the common future of humanity, empowering all people to improve their health, address health determinants and respond to health challenges”(9).

From this perspective, a key activity to be emphasized in the work plan of this CC is capacity building in nursing research, focusing on human resource development and education. Through the establishment of academic agreements with different institutions in Latin America and the Caribbean, this CC has partnered in the training of nursing professionals, aiming to strengthen the profession in these countries and promote the increase of knowledge and leadership of nurses in different areas of work(10). This is in line with SDG 17 and PAHO’s commitment to establish partnerships with CCs for capacity development, highlighting CCs “as a key element of technical cooperation offered not only by the Organization, but also among countries, as a successful example of Pan-Americanism”(8). Then, through the collaborating centers, PAHO also demonstrates its substantive participation in the achievement of SDG 17 Target 9 (Capacity Building): “Strengthen international support for the implementation of effective and targeted capacity-building in developing countries to support national plans for the implementation of all the Sustainable Development Goals, including through North-South, South-South and triangular cooperation”(8). This emphasizes the interdependence of progress in human resource training and the consequent improvement in a country’s development. It is also a form of inclusiveness that respects the needs and characteristics of each country. According to Buss, the Brazilian Cooperation Agency “defines international technical cooperation as an important instrument for development that helps countries to promote social and economic structural changes, including government policies for institutional strengthening. The programs implemented within its remit allow the transfer or sharing of knowledge, experience and best practices, developing skills and institutional capacity to promote a sustainable qualitative leap”(11).

Another aspect of capacity building is the dissemination of nursing knowledge. In addition to publishing research in major nursing journals, this CC develops free training courses for nurses in Latin America and the Caribbean. It is expected that through professional qualification, these professionals will be able to obtain better jobs and professional positions and, consequently, a possible retention of this workforce in their respective countries. This could also have an impact on the promotion of health and improved care for the population, promoting not only training aimed at the prevention and treatment of diseases, but also an increase in healthy life expectancy. It is known that healthy life expectancy involves other economic and social issues that depend on the public policies of governments, but this CC promotes initiatives that can help improve the quality of life for the community. In addition, as reiterated by Buss, global solidarity is essential, especially prioritizing the needs of the poorest and most vulnerable populations(6).

Finally, strengthening networks is essential. This CC recognizes that building partnerships is critical to developing and improving global health(12,13). The collaboration of professionals and researchers from Latin America, the Caribbean and Africa allows for the development of all those involved and enables them to find solutions to health problems together. It also allows those involved to disseminate knowledge in their countries, thus providing access to improved health for their populations. According to the PAHO/WHO document, “a regional network and official platform for the continuous sharing and cross-referencing of national experiences with the SDG goals and implementation will greatly facilitate the exchange of knowledge on sustainable development and health equity”(14). In summary, the strengthening of nursing networks will make it possible to work on an agenda that is relevant to the health problems experienced locally and regionally, to integrate different health professionals and other health-related areas in the development of research, opportunities to disseminate knowledge, collaboration with countries from Latin America, the Caribbean and Africa, and multiplying agents.

There are still many difficulties to overcome, especially in terms of establishing public government policies that could be implemented for the benefit of the population, and funding from agencies and public universities for research development and knowledge dissemination. Language is also a barrier to the dissemination of knowledge developed through collaboration, but these challenges must be faced together if these partnerships are to be established and the goals of the SDGs are to be achieved.

### Study limitations

The documents are limited to the year 2018, because at the time of the research there were no results of the activities of the CC regarding the following designation.

### Contributions to the field

In summary, the strengthening of nursing networks will make it possible to work on an agenda that is relevant to the health problems experienced locally and regionally, to integrate different health professionals and other health-related areas in the development of research, opportunities to disseminate knowledge, collaboration with countries from Latin America, the Caribbean and Africa, and multiplying agents.

## CONCLUSIONS

The analysis of the activities carried out by the Collaborating Center during these twelve years of designation concludes that it has successfully fulfilled its mission to advance the development of nursing research. This is evidenced by its processes and products in line with the SDG goals, including knowledge production, preparation of human resources for research, leadership in knowledge dissemination strategies, and transfer of research results to health services, with the aim of promoting change and strengthening nursing networks.

## Data Availability

The research data are available within the article.
