# Supplementary material for: Mission of a PAHO/WHO Collaborating Centre and the UN Sustainable Development Goals: achievements and challenges
Source: Rev Bras Enferm. 2025 Oct 27;78(Suppl 1):e20240140. doi: 10.1590/0034-7167-2024-0140 (PMC12563826; doi:10.1590/0034-7167-2024-0140)
Supplement: Translation

# Translation: Missão de um Centro Colaborador da OPAS/OMS e Objetivos de Desenvolvimento Sustentável: conquistas e desafios

**DOI:** 10.1590/0034-7167-2024-0140pt

**Authors:** Carla Aparecida Arena Ventura, Juliana Gazzotti, Isabel Amélia Costas Mendes, Pedro Fredemir Palha, Maria Helena Palucci Marziale

**Affiliations:** I Universidade de São Paulo. Ribeirão Preto, São Paulo, Brasil

**Keywords:** Desenvolvimento Sustentável, Nações Unidas, Organização Pan-Americana da Saúde, Organização Mundial da Saúde, Pesquisa em Enfermagem

## Abstract

**Objetivos:**

identificar a relação entre atividades desenvolvidas pelo Centro Colaborador e os Objetivos de Desenvolvimento Sustentável, durante os processos de redesignação de 2010, 2014 e 2018.

**Métodos:**

estudo descritivo, histórico-documental, desenvolvido entre novembro-dezembro 2023. Relatórios do Centro Colaborador foram usados como fontes primárias para coleta de dados, analisados com abordagem qualitativa. Dados foram categorizados de acordo com estruturas identificadas após organização das informações.

**Resultados:**

planos de trabalho do Centro Colaborador em 2010, 2014 e 2018 estavam relacionados aos Objetivos 3 e 4. O Plano de Trabalho de 2014 incluiu o Objetivo 12 e o de 2018 os Objetivos 8 e 17.

**Conclusões:**

o Centro Colaborador cumpriu sua missão de promover o desenvolvimento da pesquisa em enfermagem, conforme as metas dos Objetivos de Desenvolvimento Sustentável: produção de conhecimento, capacitação, disseminação do conhecimento e transferência de resultados de pesquisa para serviços de saúde, visando sua transformação, e fortalecimento das redes de enfermagem.

## INTRODUÇÃO

Centros Colaboradores (CCs) são instituições designadas pelo Diretor-Geral da Organização Mundial da Saúde (OMS) para participarem de uma rede colaborativa internacional, que apoia os programas da OMS, em todos os níveis, por meio de várias atividades. Os CCs desenvolvem atividades recomendadas pela OMS, alinhando-as com as políticas da organização e suas próprias competências(1). Em janeiro de 2000, o Conselho Executivo da OMS encorajou os Estados-Membros a utilizarem os Centros Colaboradores (CCs) da OMS como fontes valiosas de informações, serviços e conhecimentos especializados, ao mesmo tempo em que aprimoravam sua capacidade nacional de treinamento, pesquisa e colaboração no desenvolvimento da saúde(1).

Todos os Centros Colaboradores da OMS na Região das Américas são chamados de Centros Colaboradores da OPAS/OMS, pois a Organização Pan-Americana da Saúde (OPAS) funciona como o Escritório Regional da OMS e se alinha com sua estrutura constitucional. A designação é concedida por um período inicial de quatro anos e pode ser renovada antes de sua finalização(2). Como parte do processo de designação/redesignação, um Centro Colaborador da OPAS/OMS estabelece Termos de Referência que são compostos por atividades acordadas com a OMS, conforme suas políticas. Nesse contexto, as políticas da OMS estão vinculadas às metas da Organização das Nações Unidas (ONU), entre elas, os Objetivos de Desenvolvimento Sustentável (ODS). No final do século, houve mudanças na agenda de cooperação internacional, a partir do estabelecimento de metas comuns, que direcionariam os fundos e estratégias de desenvolvimento nas diferentes regiões do mundo.

Foram estabelecidas metas, bem como mecanismos de monitoramento e avaliação para analisar o alcance das metas propostas (2000-2015). “A Declaração do Milênio das Nações Unidas, assinada em setembro de 2000, comprometeu os líderes mundiais a combater a pobreza, a fome, as doenças, o analfabetismo, a degradação ambiental e a discriminação contra as mulheres”(3). Estabeleceu as oito metas dos Objetivos de Desenvolvimento do Milênio (ODM). Consequentemente, o progresso em direção aos ODM também abrangeu a redução da pobreza, melhorias na educação e maior acesso à água potável. Avanços significativos foram feitos também nas três metas e objetivos relacionados à saúde. Globalmente, as epidemias de HIV, tuberculose (TB) e malária foram substancialmente abordadas, enquanto as taxas de mortalidade infantil e materna diminuíram significativamente (em 53% e 44%, respectivamente, desde 1990), embora as metas dos ODM não tenham sido totalmente cumpridas(4).

Os ODM influenciaram significativamente as discussões globais sobre desenvolvimento e levaram a aumentos substanciais na ajuda ao desenvolvimento. No entanto, várias limitações surgiram, que incluem uma abordagem fragmentada em programas de saúde e doenças, atenção insuficiente ao fortalecimento dos sistemas de saúde, dependência de um modelo“tamanho único” para o planejamento do desenvolvimento e uma ênfase em metas agregadas em vez de resultados equitativos(5).

Tendo em vista este cenário, durante a conferência Rio+20 (Conferência das Nações Unidas sobre Desenvolvimento Sustentável) no Rio de Janeiro, em junho de 2012, a comunidade internacional iniciou um processo para criar um novo conjunto de objetivos para o desenvolvimento, visando sustentar o impulso gerado pelos ODM, e estabelecer uma estrutura de desenvolvimento global além de 2015. Em julho de 2014, o Grupo de Trabalho Aberto (OWG) da Assembleia Geral da ONU propôs um documento com 17 metas para consideração e aprovação pela Assembleia Geral, o que ocorreu em setembro de 2015. Este documento lançou as bases para os novos objetivos de desenvolvimento e a agenda de desenvolvimento global para o período de 2015 a 2030(4), denominada Agenda 2030 e composta pelos chamados ODS (Objetivos de Desenvolvimento Sustentáveis)

A Agenda de Desenvolvimento Sustentável 2030 se baseia nos ODM. Embora a erradicação da pobreza, a saúde, a educação e a segurança alimentar e nutricional ainda sejam prioridades importantes, os ODS incluem uma ampla gama de objetivos econômicos, sociais e ambientais(4).

Os 17 ODS e 169 metas da nova agenda de desenvolvimento, que inclui um objetivo específico para a saúde com 13 metas, integram as dimensões do desenvolvimento sustentável focadas nas pessoas, no planeta, na prosperidade, na paz e na parceria. O objetivo número 3 para a saúde é abrangente: “Garantir vidas saudáveis e promover o bem-estar para todos em todas as idades”(4). A saúde desempenha um papel crucial como um contribuidor-chave e beneficiário das políticas de desenvolvimento sustentável. Existem inúmeras conexões entre o objeto para a saúde e outros objetivos e metas, destacando a abordagem integrada que fundamenta os ODS. A cobertura universal de saúde (CUS), uma das 13 metas dentro do objetivo para a saúde, serve como uma estrutura abrangente para implementar uma agenda de saúde ampla e ambiciosa em todos os países. Portanto, além do ODS número 3, outros objetivos também contribuem para a promoção da saúde, reconhecendo sua importância social, ambiental e econômica, juntamente com uma abordagem multidisciplinar que inclui metas como erradicar a pobreza, garantir educação de qualidade, alcançar a igualdade de gênero e fornecer água limpa e saneamento, entre outros(4).

Nesse contexto, os CCs desempenham papel fundamental no processo descentralizado de alcance dos ODS, pois auxiliam na formação de recursos humanos, na adaptação de soluções às realidades locais, na formação de parcerias e redes que conectam diferentes setores da sociedade em busca de abordagens inovadoras e na disseminação de boas práticas(1). No Brasil, a Escola de Enfermagem de Ribeirão Preto da Universidade de São Paulo, localizada no interior do estado de São Paulo, desempenhou papel inovador e protagonista com a designação de um CC na década de 1980, o único na área de Enfermagem no país, e vem mantendo sucessivas redesignações desde então. Adicionalmente, colabora com a OPAS Brasil e o Ministério da Saúde para apoiar a implementação de políticas públicas.

## OBJETIVOS

Identificar a relação entre as atividades desenvolvidas pelo Centro Colaborador da OPAS/OMS para o Desenvolvimento da Pesquisa em Enfermagem, sediado na Escola de Enfermagem de Ribeirão Preto da Universidade de São Paulo (EERP-USP), e os Objetivos de Desenvolvimento Sustentável (ODS) da ONU, durante os processos de redesignação de 2010, 2014 e 2018.

## MÉTODOS

### Aspectos éticos

As fontes utilizadas neste estudo são de acesso público e, portanto, não necessitaram de apreciação por um Comitê de Ética em Pesquisa.

### Desenho, local e período

Trata-se de um estudo qualitativo e descritivo, de natureza histórico-documental, desenvolvido entre novembro e dezembro de 2023, na EERP-USP. Os dados foram coletados a partir de nove relatórios anuais do Centro Colaborador enviados à OMS. Os relatórios anuais são solicitados automaticamente pela OMS por e-mail e são relativos aos últimos doze meses da sua designação anterior. Para este estudo, nove relatórios anuais (de 2010 a 2018) foram utilizados para coleta de dados.

### População e amostra

Foram utilizadas fontes primárias, ou seja, processos e relatórios elaborados na EERP-USP em 2010, 2014 e 2018. Vale destacar que em 1988, a EERP-USP foi designada pela primeira vez como Centro Colaborador da Organização Mundial da Saúde (OMS) para o Desenvolvimento da Pesquisa em Enfermagem. Após isso, a EERP-USP foi redesignada em 1992, 1997, 2002, 2006, 2010, 2014 e 2018 por um período de 4 anos em cada redesignação e, desde então, vem trabalhando alinhada aos ODS. A última redesignação foi em 2022 e durará até 2026. Esta última redesignação não foi incluída, pois alguns dos Termos de Referência (ToRs) foram parcialmente modificados.

### Protocolo do estudo

Foi desenvolvido roteiro que envolveu a identificação e localização de fontes, análise dos relatórios e estabelecimento da conexão das atividades desenvolvidas pelo CC aos ODS.

### Análise dos resultados

Foram realizadas análises descritivas das atividades contidas nos ToRs, que são reportados anualmente à OMS. A partir disso, foi verificado se as atividades realizadas pelo CC contemplavam as metas dos ODS. Dois pesquisadores realizaram a análise de forma independente e, em caso de divergência na relação entre atividades e metas, um terceiro pesquisador foi chamado para auxiliar na obtenção de um consenso.

Os achados foram classificados com base nas estruturas relevantes identificadas, após a organização das informações. Em 2010, o CC tinha 3 Termos de Referência (ToRs) e 11 atividades (2 relacionadas ao ToR1, 7 relacionadas ao ToR2 e 2 vinculadas ao ToR3) e em 2014, o CC tinha 3 ToRs e 9 atividades (3 relacionadas ao ToR1, 4 relacionadas ao ToR2 e 2 relacionadas ao ToR3). Em 2018, o CC tinha 3 Termos de Referência (ToRs) e 10 atividades (3 relacionadas ao ToR1, 4 relacionadas ao ToR2 e 3 vinculadas ao ToR3).

## RESULTADOS

Termos de Referência (redesignação de 2010, 2014 e 2018):

TR#1 Colaborar com a OPAS/OMS no fortalecimento da pesquisa em enfermagem para o desenvolvimento de recursos humanos e da prática em enfermagem nas áreas prioritárias.

TR#2 Colaborar com a OPAS/OMS no fortalecimento e expansão da disseminação da informação sobre saúde e conhecimento com ênfase em enfermagem e obstetrícia.

TR#3 Colaborar com a OPAS/OMS para fortalecer o desenvolvimento de docentes em enfermagem através da formação e atualização em pesquisa para melhorar a qualidade científica e o currículo de enfermagem.

Os resultados estão sintetizados no Quadro 1.

**Quadro 1:** Objetivos, Metas e Atividades desenvolvidas pelo Centro Colaborador

OBJETIVOS*	METAS*	Atividades desenvolvidas pelo CC
Objetivo 3. Assegurar uma vida saudável e promover o bemestar para todas e todos, em todas as idades.	3.1 Até 2030, reduzir a taxa de mortalidade materna global para menos de 70 mortes por 100.000 nascidos vivos.	O CC desenvolveu estratégias relacionadas à iniciativa para alcançar a Maternidade Segura na América Latina e no Caribe. Como parceira dessa iniciativa, duas integrantes do CC, especialistas em Saúde da Mulher, participaram da Aliança Global para Enfermagem e Obstetrícia – GANM/OPAS para o desenvolvimento de liderança em obstetrícia, em forças-tarefa para alcançar a Maternidade Segura, assumindo a posição de liderança/facilitadora da *Community Practice Nursing and Midwifery for Making Pregnancy Safer*, especialmente em países de língua portuguesa e espanhola.
Objetivo 3. Assegurar uma vida saudável e promover o bemestar para todas e todos, em todas as idades.	3.1 Até 2030, reduzir a taxa de mortalidade materna global para menos de 70 mortes por 100.000 nascidos vivos.	O CC participou do desenvolvimento de um projeto de pesquisa colaborativa em parceria com a University of Alberta. O projeto intitulado “*Immigrant and Aboriginal Women’s Food Choice and Practice*” analisou como as crenças e hábitos de saúde de mulheres imigrantes e aborígenes, durante a gravidez, influenciam suas escolhas alimentares e como essas escolhas, por sua vez, afetam sua saúde geral.
Objetivo 3. Assegurar uma vida saudável e promover o bemestar para todas e todos, em todas as idades.	3.2 Até 2030, acabar com as mortes evitáveis de recém-nascidos e crianças menores de 5 anos, com todos os países objetivando reduzir a mortalidade neonatal para pelo menos 12 por 1.000 nascidos vivos e a mortalidade de crianças menores de 5 anos para pelo menos 25 por 1.000 nascidos vivos.	O CC participou do desenvolvimento de um projeto de pesquisa multicêntrico para ensinar a prestação de cuidados em escolas de enfermagem na América Latina e no Caribe, a fim de prevenir doenças infantis.
Objetivo 3. Assegurar uma vida saudável e promover o bemestar para todas e todos, em todas as idades.	3.2 Até 2030, acabar com as mortes evitáveis de recém-nascidos e crianças menores de 5 anos, com todos os países objetivando reduzir a mortalidade neonatal para pelo menos 12 por 1.000 nascidos vivos e a mortalidade de crianças menores de 5 anos para pelo menos 25 por 1.000 nascidos vivos.	O CC participou do desenvolvimento de um projeto de pesquisa multicêntrico financiado pelas Agências de Amparo à Pesquisa do Governo Federal e Estadual do Brasil – Fundação Bill & Melinda Gates – *Fogarty International Center*, que teve como objetivo implementar e avaliar a eficácia na implementação da Iniciativa Hospital Amigo da Criança para unidades neonatais.
Objetivo 3. Assegurar uma vida saudável e promover o bemestar para todas e todos, em todas as idades.	3.2 Até 2030, acabar com as mortes evitáveis de recém-nascidos e crianças menores de 5 anos, com todos os países objetivando reduzir a mortalidade neonatal para pelo menos 12 por 1.000 nascidos vivos e a mortalidade de crianças menores de 5 anos para pelo menos 25 por 1.000 nascidos vivos.	O CC participou do desenvolvimento de uma pesquisa conjunta com a *Florence Nightingale School of Nursing and Midwifery-King’s College London* sobre necessidades essenciais e vulnerabilidades no cuidado infantil no contexto da atenção primária à saúde. Um membro do CC foi um dos coordenadores deste projeto, que resultou em um artigo publicado intitulado “Segurança infantil na perspectiva das necessidades essenciais”.
Objetivo 3. Assegurar uma vida saudável e promover o bemestar para todas e todos, em todas as idades.	3.2 Até 2030, acabar com as mortes evitáveis de recém-nascidos e crianças menores de 5 anos, com todos os países objetivando reduzir a mortalidade neonatal para pelo menos 12 por 1.000 nascidos vivos e a mortalidade de crianças menores de 5 anos para pelo menos 25 por 1.000 nascidos vivos.	O CC participou de um projeto de pesquisa colaborativa que teve como objetivo aprimorar um objeto digital de aprendizagem (e-baby) a fim de incluir um design emocional para avaliar o impacto de seu uso nas reações emocionais de estudantes de enfermagem brasileiros e portugueses. O projeto foi desenvolvido em parceria com a Escola Superior de Enfermagem de Coimbra, Portugal.
Objetivo 3. Assegurar uma vida saudável e promover o bemestar para todas e todos, em todas as idades.	3.2 Até 2030, acabar com as mortes evitáveis de recém-nascidos e crianças menores de 5 anos, com todos os países objetivando reduzir a mortalidade neonatal para pelo menos 12 por 1.000 nascidos vivos e a mortalidade de crianças menores de 5 anos para pelo menos 25 por 1.000 nascidos vivos.	Este CC desenvolveu um projeto de pesquisa multicêntrico em parceria com a Universidade Federal da Bahia, Instituto Índio de Saúde Pública e London School of Hygiene and Tropical Medicine intitulado “Efeitos das manifestações neurológicas congênitas associadas ao Zika vírus sobre o desenvolvimento cognitivo infantil: um estudo de coorte prospectiva no contexto da Atenção Básica, em Salvador-BA”para avaliar o espectro de resultados neurocognitivos até os primeiros três anos e meio, determinando diferenças de desempenho entre crianças expostas e não expostas às consequências neurológicas da infecção materna pelo vírus Zika durante a gestação.
Objetivo 3. Assegurar uma vida saudável e promover o bemestar para todas e todos, em todas as idades.	3.2 Até 2030, acabar com as mortes evitáveis de recém-nascidos e crianças menores de 5 anos, com todos os países objetivando reduzir a mortalidade neonatal para pelo menos 12 por 1.000 nascidos vivos e a mortalidade de crianças menores de 5 anos para pelo menos 25 por 1.000 nascidos vivos.	O CC desenvolveu uma pesquisa multicêntrica em parceria com a Faculdade de Economia, Administração e Contabilidade de Ribeirão Preto da Universidade de São Paulo (FEARP-USP) e o Departamento de Estudos da Criança, Juventude e Família da Faculdade de Educação e Ciências Humanas da Universidade de Nebraska-Lincoln, EUA intitulada “Escolha do tipo de cuidado para criança pequena: desenvolvimento infantil e necessidades familiares”. Este estudo mapeou como as famílias planejam os cuidados para o futuro bebê, como esse planejamento foi alterado após o nascimento do bebê e o quanto os serviços de cuidado para a criança estavam de acordo com as necessidades da criança e da família.
Objetivo 3. Assegurar uma vida saudável e promover o bemestar para todas e todos, em todas as idades.	3.2 Até 2030, acabar com as mortes evitáveis de recém-nascidos e crianças menores de 5 anos, com todos os países objetivando reduzir a mortalidade neonatal para pelo menos 12 por 1.000 nascidos vivos e a mortalidade de crianças menores de 5 anos para pelo menos 25 por 1.000 nascidos vivos.	O CC desenvolveu uma pesquisa colaborativa com a Western Sydney University, Austrália. O objetivo foi avaliar a eficácia da acupuntura atrial em mulheres lactantes para melhorar as condições de amamentação e aumentar a produção diária de leite.
Objetivo 3. Assegurar uma vida saudável e promover o bemestar para todas e todos, em todas as idades.	3.3 Até 2030, acabar com as epidemias de AIDS, tuberculose, malária e doenças tropicais negligenciadas, e combater a hepatite, doenças transmitidas pela água, e outras doenças transmissíveis	O CC integrou redes internacionais e nacionais nas áreas de tuberculose e TB/HIV e coordenou projetos de pesquisa no Brasil (nordeste, sul e sudeste do país e pesquisas na África) sobre avaliação da atenção primária no tratamento da tuberculose, gestão dos serviços de saúde e ações de Enfermagem no controle da tuberculose na atenção primária e transferência de políticas sobre controle da tuberculose.
Objetivo 3. Assegurar uma vida saudável e promover o bemestar para todas e todos, em todas as idades.	3.5 Reforçar a prevenção e o tratamento do abuso de substâncias, incluindo o abuso de drogas entorpecentes e uso nocivo do álcool.	O CC participou do desenvolvimento de um projeto de pesquisa multicêntrico destinado a investigar as consequências do consumo de drogas entre estudantes de graduação em nove universidades de seis países latinoamericanos e três países caribenhos. As universidades envolvidas foram Escola de Enfermagem de Ribeirão Preto da Universidade de São Paulo, Universidade de Concepción, Universidade da Costa Rica, Universidade Evangélica de El Salvador, Universidade do Caribe Norte, Universidade Benemérita Autônoma de Puebla, Universidade Nacional Autônoma de Nicarágua, Universidade Anton de Kon do Suriname e Universidade do Sul do Caribe. O estudo utilizou o mesmo instrumento de pesquisa contendo 70 itens modificados com questões relacionadas às características sociodemográficas e conhecimento dos efeitos do álcool, maconha e cocaína relacionados ao desempenho acadêmico.
Objetivo 3. Assegurar uma vida saudável e promover o bemestar para todas e todos, em todas as idades.	3.5 Reforçar a prevenção e o tratamento do abuso de substâncias, incluindo o abuso de drogas entorpecentes e uso nocivo do álcool.	O CC faz parte do Comitê Organizador do Encontro bianual de Pesquisadores em Saúde Mental e Especialistas em Enfermagem Psiquiátrica. Esses encontros têm como objetivo refletir sobre para onde a área de saúde mental está caminhando em termos de assistência, ensino e pesquisa, por meio da apresentação de estudos de profissionais e acadêmicos dessa área.
Objetivo 3. Assegurar uma vida saudável e promover o bemestar para todas e todos, em todas as idades.	3.8 Atingir a cobertura universal de saúde, incluindo a proteção do risco financeiro, o acesso a serviços de saúde essenciais de qualidade e o acesso a medicamentos e vacinas essenciais seguros, eficazes, de qualidade e a preços acessíveis para todos.	Uma das mudanças necessárias na educação em enfermagem é aumentar o número de enfermeiros na América Latina e Caribe com títulos de doutorado. Isso ajudará a desenvolver profissionais para liderar o avanço científico da área de enfermagem. Em dezembro de 2016, em parceria com a OPAS, membros deste CC foram responsáveis por revisar e fazer sugestões ao documento “Plano de Ação para Programas de Doutorado na América Latina e Caribe”. O objetivo do documento foi propor estratégias para promover a formação de alunos de doutorado na América Latina e Caribe por meio da cooperação técnica, acadêmica e de pesquisa e do desenvolvimento de programas de pós-graduação para a geração e transferência de conhecimento e inovação tecnológica para contribuir para a transformação da realidade de saúde dos países. Além disso, em 2017 houve o lançamento do documento “*Formación doctoral en enfermeira en América Latina y el Caribe*”, que fornece uma análise dos programas de doutorado na América Latina e Caribe, acompanhada de um plano de ação.
Objetivo 3. Assegurar uma vida saudável e promover o bemestar para todas e todos, em todas as idades.	3.8 Atingir a cobertura universal de saúde, incluindo a proteção do risco financeiro, o acesso a serviços de saúde essenciais de qualidade e o acesso a medicamentos e vacinas essenciais seguros, eficazes, de qualidade e a preços acessíveis para todos.	O CC foi responsável por desenvolver um compêndio de estudos de caso brasileiros exemplares, que mostram as melhores práticas em enfermagem, visando promover o acesso universal à saúde e a cobertura universal de saúde. Os membros deste CC desenvolvem projetos de pesquisa visando alcançar o acesso universal à saúde nas Américas, cujos resultados estão publicados no site do CC.
Objetivo 3. Assegurar uma vida saudável e promover o bemestar para todas e todos, em todas as idades.	3.C Aumentar substancialmente o financiamento da saúde e o recrutamento, desenvolvimento e formação, e retenção do pessoal de saúde nos países em desenvolvimento, especialmente nos países menos desenvolvidos e nos pequenos Estados insulares em desenvolvimento.	O CC participou do Programa de Cooperação Internacional em Saúde Brasil-Uruguai, apoiado pelo Ministério da Saúde do Brasil e pela OPAS. Foram desenvolvidos os seguintes projetos de pesquisa: “Projeto de Cooperação Internacional em Saúde: Transferência de tecnologia de enfermagem em pesquisas censitárias” para apoiar a troca de experiências, conhecimentos e tecnologias relacionadas à estruturação e desenvolvimento de estudo censitário sobre a força de trabalho de enfermagem no Uruguai; “Dimensionamento da força de trabalho: classificação das práticas em atenção primária à saúde”; “Método de dimensionamento da força de trabalho na Atenção Primária à Saúde”; e “Dimensionamento de pessoal e caracterização de competências da atenção primária à saúde para prática colaborativa”.
Objetivo 3. Assegurar uma vida saudável e promover o bemestar para todas e todos, em todas as idades.	3.C Aumentar substancialmente o financiamento da saúde e o recrutamento, desenvolvimento e formação, e retenção do pessoal de saúde nos países em desenvolvimento, especialmente nos países menos desenvolvidos e nos pequenos Estados insulares em desenvolvimento.	O CC desenvolveu um projeto de pesquisa intitulado “Desenvolvimento de infográfico animado sobre intervenções obstétricas durante o trabalho de parto”, que teve como objetivos revisar a literatura científica sobre as intervenções obstétricas recomendadas, os procedimentos em que os benefícios e danos devem ser avaliados e os procedimentos que são realizados durante as fases do trabalho de parto; desenvolver um infográfico animado sobre intervenções durante o trabalho de parto; revisar e validar o infográfico animado desenvolvido; e avaliar a capacidade do infográfico de estimular o aprendizado dos profissionais de saúde relacionados às intervenções durante o trabalho de parto.
Objetivo 3. Assegurar uma vida saudável e promover o bemestar para todas e todos, em todas as idades.	3.C Aumentar substancialmente o financiamento da saúde e o recrutamento, desenvolvimento e formação, e retenção do pessoal de saúde nos países em desenvolvimento, especialmente nos países menos desenvolvidos e nos pequenos Estados insulares em desenvolvimento.	O CC desenvolveu um projeto de pesquisa intitulado “Laboratório virtual para desenvolvimento de competências e iniciativas em empreendedorismo de alto impacto social”, com o intuito desenvolver e avaliar a qualidade e usabilidade de um jogo (protótipo) voltado para empreendedorismo e inovação de alto impacto social. O objetivo foi promover a disseminação do conhecimento e fortalecer uma cultura de inovação e empreendedorismo, incentivando a incorporação e aplicação desses princípios na identificação de problemas e soluções para a assistência à saúde, educação e gestão em saúde e enfermagem. Por meio de atividades on-line e da disponibilização de materiais digitais informativos e educativos, o jogo serviu como um espaço virtual para simulação e experimentação, transformando ideias inovadoras em novos processos, produtos, serviços e negócios de alto impacto social.
Objetivo 3. Assegurar uma vida saudável e promover o bemestar para todas e todos, em todas as idades.	3.C Aumentar substancialmente o financiamento da saúde e o recrutamento, desenvolvimento e formação, e retenção do pessoal de saúde nos países em desenvolvimento, especialmente nos países menos desenvolvidos e nos pequenos Estados insulares em desenvolvimento.	O CC também desenvolveu um projeto de pesquisa sobre a prática colaborativa interprofissional na formação em enfermagem no Brasil com o objetivo de analisar a formação de enfermeiros quanto às práticas colaborativas interprofissionais; analisar os projetos político-pedagógicos e os currículos dos cursos de enfermagem no Brasil, identificando o desenvolvimento de ações interprofissionais; e identificar aspectos facilitadores e dificultadores da formação interprofissional de enfermeiros.
Objetivo 3. Assegurar uma vida saudável e promover o bemestar para todas e todos, em todas as idades.	3.D Reforçar a capacidade de todos os países, particularmente os países em desenvolvimento, para o alerta precoce, redução de riscos e gerenciamento de riscos nacionais e globais de saúde.	O CC participou de um projeto de pesquisa multicêntrico em parceria com a Escola de Enfermagem da Universidade do Alabama em Birmingham, EUA e a Universidad Nacional Autónoma de México, México, para identificar competências de enfermagem em Saúde Global. O objetivo foi validar o questionário “*Global Health Competency Survey*” para a cultura brasileira e identificar o grau de concordância dos docentes de enfermagem brasileiros em relação às competências em saúde global a serem adquiridas pelo aluno durante o curso de graduação em enfermagem.
Objetivo 4. Assegurar a educação inclusiva e equitativa e de qualidade, e promover oportunidades de aprendizagem ao longo da vida para todas e todos.	4.3 Até 2030, assegurar a igualdade de acesso para todos os homens e mulheres à educação técnica, profissional e superior de qualidade, a preços acessíveis, incluindo universidade.	O CC colabora com a OPAS na organização e manutenção do Diretório PanAmericano de Escolas de Enfermagem. Foi realizada uma extensa busca para alimentar o banco de dados, que visa reunir informações de Instituições de Enfermagem, disponibilizando-as ao público em geral, a fim de aumentar a visibilidade das escolas de enfermagem e fornecer uma gama de opções de escolas reconhecidas que oferecem educação acessível e de qualidade.
Objetivo 4. Assegurar a educação inclusiva e equitativa e de qualidade, e promover oportunidades de aprendizagem ao longo da vida para todas e todos.	4.4 Até 2030, aumentar substancialmente o número de jovens e adultos que tenham habilidades relevantes, inclusive competências técnicas e profissionais, para emprego, trabalho decente e empreendedorismo.	O CC participou de um projeto de pesquisa conjunto focado na simulação como estratégia de ensino para o ensino de enfermagem. Este projeto foi desenvolvido em parceria com a Escola Superior de Enfermagem de Coimbra e analisou evidências científicas relacionadas à simulação no ensino de enfermagem, com o objetivo de validar materiais pedagógicos de acordo com os aspectos culturais, sociais e educacionais, melhorar experiências clínicas com base no desenvolvimento de competências clínicas, relacionamento, ética e pensamento crítico, analisar o processo de implementação de experiências de aprendizagem simuladas na perspectiva de professores e alunos, e analisar o que eles aprenderam durante essas experiências simuladas.
Objetivo 4. Assegurar a educação inclusiva e equitativa e de qualidade, e promover oportunidades de aprendizagem ao longo da vida para todas e todos.	4.4 Até 2030, aumentar substancialmente o número de jovens e adultos que tenham habilidades relevantes, inclusive competências técnicas e profissionais, para emprego, trabalho decente e empreendedorismo.	O CC estabeleceu uma parceria com o Ministério da Saúde do Brasil para a formação de recursos humanos em saúde no Haiti.
Objetivo 4. Assegurar a educação inclusiva e equitativa e de qualidade, e promover oportunidades de aprendizagem ao longo da vida para todas e todos.	4.4 Até 2030, aumentar substancialmente o número de jovens e adultos que tenham habilidades relevantes, inclusive competências técnicas e profissionais, para emprego, trabalho decente e empreendedorismo.	Um projeto de educação continuada em saúde foi desenvolvido por este CC para fortalecer a atenção primária. O projeto foi realizado em vinte e quatro cidades e envolveu mais de cem trabalhadores de diferentes formações, com participação de pesquisadores e gestores de diferentes instituições. Entre as atividades, destaca-se a participação de pesquisadores em um curso organizado por instituições brasileiras e norte-americanas, que promoveu pesquisa e formação na área de economia do trabalho e educação em saúde para pesquisadores do Mercosul e da Região Andina.
Objetivo 4. Assegurar a educação inclusiva e equitativa e de qualidade, e promover oportunidades de aprendizagem ao longo da vida para todas e todos.	4.7 Até 2030, garantir que todos os alunos adquiram conhecimentos e habilidades necessárias para promover o desenvolvimento sustentável, inclusive, entre outros, por meio da educação para o desenvolvimento sustentável e estilos de vida sustentáveis, direitos humanos, igualdade de gênero, promoção de uma cultura de paz e não violência, cidadania global e valorização da diversidade cultural e da contribuição da cultura para o desenvolvimento sustentável.	O CC participou de um projeto colaborativo em parceria com a University of Surrey. O objetivo era comparar legislações do Brasil e do Reino Unido sobre direitos humanos de pacientes durante internação involuntária em hospitais psiquiátricos com base na Lista de Verificação da OMS sobre Legislações de Saúde Mental.
Objetivo 4. Assegurar a educação inclusiva e equitativa e de qualidade, e promover oportunidades de aprendizagem ao longo da vida para todas e todos.	4.7 Até 2030, garantir que todos os alunos adquiram conhecimentos e habilidades necessárias para promover o desenvolvimento sustentável, inclusive, entre outros, por meio da educação para o desenvolvimento sustentável e estilos de vida sustentáveis, direitos humanos, igualdade de gênero, promoção de uma cultura de paz e não violência, cidadania global e valorização da diversidade cultural e da contribuição da cultura para o desenvolvimento sustentável.	O CC desenvolveu um projeto de pesquisa colaborativa com a Faculdade de Enfermagem da Universidade de Alberta e o John Dossetor Health Ethics Centre, em Edmonton, Canadá. O objetivo foi explorar a relação entre saúde mental, direitos humanos e ética; entender os mecanismos e procedimentos de proteção para indivíduos com transtornos de saúde mental internados em hospitais psiquiátricos no Canadá; comparar os mecanismos e procedimentos de proteção de direitos humanos para pessoas com doenças mentais no Canadá com aqueles no Brasil; e desenvolver um programa de treinamento focado em direitos humanos e ética em cuidados de saúde mental para profissionais de saúde.
Objetivo 4. Assegurar a educação inclusiva e equitativa e de qualidade, e promover oportunidades de aprendizagem ao longo da vida para todas e todos.	4.B Até 2020, substancialmente ampliar globalmente o número de bolsas de estudo para os países em desenvolvimento, em particular os países menos desenvolvidos, pequenos Estados insulares em desenvolvimento e os países africanos, para o ensino superior, incluindo programas de formação profissional, de tecnologia da informação e da comunicação, técnicos, de engenharia e programas científicos em países desenvolvidos e outros países em desenvolvimento.	O CC recebeu enfermeiras de Moçambique por meio do Programa “Mujeres por Africa”. Este Programa foi formalizado por um Acordo entre Brasil, Espanha e África. As enfermeiras foram matriculadas em um treinamento profissional e educação continuada e desenvolveram atividades de prática clínica e aprendizado teórico em diferentes áreas, como saúde da mulher, assistência integral à saúde da criança e do adolescente, avaliação de exames médicos, tratamento de feridas, câncer de mama e ortopedia, entre outras. O programa contribuiu para o desenvolvimento de recursos humanos em Moçambique, aprimorando as habilidades e competências profissionais dessas enfermeiras.
Objetivo 4. Assegurar a educação inclusiva e equitativa e de qualidade, e promover oportunidades de aprendizagem ao longo da vida para todas e todos.	4.B Até 2020, substancialmente ampliar globalmente o número de bolsas de estudo para os países em desenvolvimento, em particular os países menos desenvolvidos, pequenos Estados insulares em desenvolvimento e os países africanos, para o ensino superior, incluindo programas de formação profissional, de tecnologia da informação e da comunicação, técnicos, de engenharia e programas científicos em países desenvolvidos e outros países em desenvolvimento. O CC recebia anualmente uma delegação de estudantes de graduação em enfermagem da Universidade Agostinho Neto, Angola, para um período de estágio clínico de três meses.	Os estudantes eram financiados por esta parceria e participavam de cursos nas seguintes áreas: enfermagem materno-infantil, doenças, drogas e álcool, visando sanar as dificuldades mais urgentes do país enfrentadas na área da saúde.
Objetivo 4. Assegurar a educação inclusiva e equitativa e de qualidade, e promover oportunidades de aprendizagem ao longo da vida para todas e todos.	4.B Até 2020, substancialmente ampliar globalmente o número de bolsas de estudo para os países em desenvolvimento, em particular os países menos desenvolvidos, pequenos Estados insulares em desenvolvimento e os países africanos, para o ensino superior, incluindo programas de formação profissional, de tecnologia da informação e da comunicação, técnicos, de engenharia e programas científicos em países desenvolvidos e outros países em desenvolvimento.	O CC também teve uma parceria com a Universidade Agostinho Neto por meio da implementação de um Mestrado em Enfermagem e Obstetrícia no país. Dentro deste programa, os membros deste CC ofereciam cursos presenciais e visitavam periodicamente a Universidade Agostinho Neto como parte de sua função de gestão em relação ao programa.
Objetivo 4. Assegurar a educação inclusiva e equitativa e de qualidade, e promover oportunidades de aprendizagem ao longo da vida para todas e todos.	4.C Até 2030, substancialmente aumentar o contingente de professores qualificados, inclusive por meio da cooperação internacional para a formação de professores, nos países em desenvolvimento, especialmente os países menos desenvolvidos e pequenos Estados insulares em desenvolvimento.	O CC participou do desenvolvimento de um projeto de pesquisa multicêntrico em parceria com a Université de Cergy Pontoise, França, para examinar a promoção da saúde e a prevenção de doenças na educação profissional de enfermagem em ambos os países.
Objetivo 4. Assegurar a educação inclusiva e equitativa e de qualidade, e promover oportunidades de aprendizagem ao longo da vida para todas e todos.	4.C Até 2030, substancialmente aumentar o contingente de professores qualificados, inclusive por meio da cooperação internacional para a formação de professores, nos países em desenvolvimento, especialmente os países menos desenvolvidos e pequenos Estados insulares em desenvolvimento.	O CC desenvolveu um curso virtual intitulado “Metodologia Científica e Estratégias para Disseminação do Conhecimento”, oferecido em português, espanhol e inglês. Este curso visa aprimorar a compreensão do desenvolvimento de pesquisa em saúde e enfermagem. É um curso online aberto, com as versões em espanhol e inglês disponíveis no Campus Virtual de Saúde Pública (VCPH/OPAS).
Objetivo 8. Promover o crescimento econômico sustentado, inclusivo e sustentável, emprego pleno e produtivo e trabalho decente para todas e todos	8.5 Até 2030, alcançar o emprego pleno e produtivo e trabalho decente para todas as mulheres e homens, inclusive para os jovens e as pessoas com deficiência, e remuneração igual para trabalho de igual valor.	O CC oferece cursos de pós-graduação e desenvolve estudos na área de Saúde Ocupacional que capacitam mestrandos e doutorandos para realizar ações e contribuir para a promoção de ambientes de trabalho saudáveis por meio da identificação e intervenção em situações de risco ocupacional. Com o advento da pandemia de covid-19, em 2021 o CC participou de um projeto de pesquisa em parceria com a OPAS/OMS intitulado “Issue Brief: Impactos da COVID-19 nos profissionais de saúde e estratégias utilizadas para mitigar o problema no Brasil”cujo objetivo foi analisar o impacto da covid-19 nas condições de trabalho e infecção por covid-19 em profissionais de saúde no Brasil durante a pandemia com base em dados epidemiológicos e evidências científicas. Os dados coletados foram enviados à OMS e incluídos em seus relatórios internacionais de indicadores. No Brasil, também foi apresentado a gestores e formuladores de políticas públicas durante o “Webinar Internacional: impactos da covid-19 nas condições de trabalho e saúde dos profissionais de saúde no Brasil”promovido por este CC em parceria com o Ministério da Saúde e a OPAS/Brasil.
Objetivo 12. Assegurar padrões de produção e de consumo sustentáveis	12.4 Até 2020, alcançar o manejo ambientalmente saudável dos produtos químicos e todos os resíduos, ao longo de todo o ciclo de vida destes, de acordo com os marcos internacionais acordados, e reduzir significativamente a liberação destes para o ar, água e solo, para minimizar seus impactos negativos sobre a saúde humana e o meio ambiente.	O CC participou de um projeto de pesquisa conjunto sobre a ferramenta de gestão ambiental para avaliação de risco em bacias hidrográficas. O projeto recebeu financiamento da Agência Espanhola de Financiamento - Programa de Cooperación Interuniversitaria e Investigación Científica (PCI) foi desenvolvido em parceria com a Universitat Rovira i Virgili, Espanha.
Objetivo 17. Fortalecer os meios de implementação e revitalizar a parceria global para o desenvolvimento sustentável.	17.9 Reforçar o apoio internacional para a implementação eficaz e orientada da capacitação em países em desenvolvimento, a fim de apoiar os planos nacionais para implementar todos os objetivos de desenvolvimento sustentável, inclusive por meio da cooperação NorteSul, Sul-Sul e triangular.	O CC foi contatado pelo Assessor Regional de Enfermagem e Pessoal de Saúde Aliado/Sistemas e Serviços de Saúde da Organização Pan-Americana da Saúde (OPAS/OMS) para colaborar com o Ministério da Saúde Pública da Guiana, que tinha o objetivo de desenvolver a capacitação de Recursos Humanos em Enfermagem e Obstetrícia para a Guiana, a fim de mover a Educação em Enfermagem e a Prática de Enfermagem para os níveis de Mestrado e Doutorado. Um Plano de Trabalho e um acordo foram desenvolvidos e aprovados por ambas as instituições. O edital para o processo de seleção foi lançado em abril de 2018 e a Diretora do CC viajou para a Guiana para participar do Comitê responsável pela seleção de 13 alunos, oito para o Programa de Mestrado e cinco para o Programa de Doutorado. Os alunos iniciaram seus programas de pós-graduação em setembro de 2018. Esta atividade é integrada ao objetivo abrangente de promover o desenvolvimento e o fortalecimento da pesquisa em enfermagem na América Latina e no Caribe por meio do desenvolvimento e capacitação de recursos humanos.

*OBS: Os Objetivos e Metas dos ODS estão escritos exatamente de acordo com a referência 4.

## DISCUSSÃO

Os Centros Colaboradores (CCs) da Organização Mundial da Saúde (OMS) são vitais para o avanço das iniciativas globais de saúde. Estes Centros, que incluem instituições de pesquisa, universidades e outras entidades designadas, são encarregados de atividades específicas que dão suporte aos programas estratégicos da OMS. Suas contribuições são cruciais para a implementação eficaz de metas globais de saúde, como os Objetivos de Desenvolvimento Sustentável dos ODS da ONU, particularmente o ODS 3, que visa assegurar uma vida saudável e promover o bem-estar para todos em todas as idades. Suas principais funções incluem o desenvolvimento de pesquisas que abordem questões prioritárias de saúde; desenvolver e disseminar conhecimento; capacitar recursos humanos; e implementar agendas e emergências globais de saúde(1). Em vista disso, os planos de trabalho do CC em 2010, 2014 e 2018 estava principalmente relacionado aos ODS 3 e 4, “Assegurar uma vida saudável e promover o bem-estar para todos em todas as idades”(4) e “Assegurar a educação inclusiva e equitativa e de qualidade, e promover oportunidades de aprendizagem ao longo da vida para todas e todos”(4). O Plano de Trabalho de 2014 também incluiu o ODS 12 “Assegurar padrões de produção e de consumo sustentáveis”(4) e o Plano de Trabalho de 2018 também incluiu os ODS 8 “Promover o crescimento econômico sustentado, inclusivo e sustentável, emprego pleno e produtivo e trabalho decente para todas e todos”(4) e 17 “Fortalecer os meios de implementação e revitalizar a parceria global para o desenvolvimento sustentável”(4). Ressalta-se que, em relação ao ODS 8, este CC promove a capacitação de recursos humanos ao desenvolver ações e contribuir para a promoção de ambientes de trabalho saudáveis, por meio da identificação e intervenção em situações de riscos ocupacionais. Além disso, durante a pandemia da covid-19, o CC em parceria com a OPAS/OMS e o Ministério da Saúde do Brasil, identificou indicadores de risco de infecção pelo SARS-Cov-2 e adoecimento e mortes por covid-19 entre profissionais de saúde.

Considerando as metas, em 2010, as atividades estavam relacionadas às metas 3.1 (2), 3.2 (4), 3.3, 3.5, 3.C, 4.3, 4.4, 4.7 e 4.B (3). Em 2014, estavam relacionadas às metas 3.2 (3), 3.5, 3.8, 3. D, 4.4 (2), 4.C e 12.4. Em 2018, estavam relacionadas às metas 3.8, 3.C (3), 4.7, 4.C e 17.9.

As atividades do CC relacionadas aos ODS foram agrupadas em quatro categorias:

1. Atividades de pesquisa com foco em saúde materna, saúde infantil, doenças transmissíveis, prevenção e tratamento e saúde mental;
2. Fortalecimento da pesquisa em enfermagem por meio do desenvolvimento de recursos humanos e avanço educacional;
3. Disseminação do conhecimento em enfermagem; e
4. Fortalecimento das redes de enfermagem.

Dentre as metas dos ODS, as da área da saúde são fundamentais para o desenvolvimento global, especialmente das populações mais vulneráveis. Segundo Buss, há consenso entre autores de que a saúde,“entendida como populações saudáveis e não apenas a ausência de doenças, é fundamental para o desenvolvimento econômico e social”(6). Ao mesmo tempo, o ODS 3“Assegurar uma vida saudável e promover o bem-estar para todas e todos, em todas as idades” é “afetado pela mensuração dos resultados de muitos outros ODS econômicos, sociais e ambientais”(7).

Como observado no agrupamento de atividades desenvolvidas por este CC, a maioria delas está relacionada ao ODS 3, demonstrando o foco na prevenção e no cuidado integrado e centrado no paciente. Os projetos de pesquisa realizados por este CC são importantes ferramentas para auxiliar na redução, controle e tratamento de doenças, estando, portanto, em concordância com o estabelecido pelos ODS e a favor do alcance de suas metas(3). Este trabalho também é desenvolvido em convergência com os objetivos da OPAS/OMS(2). Segundo a OPAS, essa convergência fortalece a função estratégica dos Centros Colaboradores, em particular para a realização do ODS 3.“Dado seu alcance global e o foco que muitos deles colocam na capacitação nos níveis local, regional e global, esses centros oferecem instrumentos-chave para a realização do ODS 3 e outras metas futuras”(8). Essas atividades estão incluídas nos relatórios anuais do CC enviados à OMS.

A OPAS/OMS tem se esforçado para implementar a Agenda 2030. Um documento importante para isso é o 13º Plano Geral deTrabalho da Organização Mundial da Saúde, aprovado em maio de 2018, na 71ª Assembleia Mundial da Saúde. O documento teve como objetivo estabelecer condições para melhorar a saúde das populações nos próximos cinco anos. A OMS reconheceu que o sucesso desse documento dependeria do alinhamento de seus esforços com os ODS. “O GPW 13 é baseado nos ODS e é relevante para todos os países – baixa, média e alta renda. A saúde é fundamental para os ODS e, em um mundo interconectado, o papel da OMS em fornecer bens públicos globais, que ajudem a garantir a saúde para todas as pessoas dentro e além das fronteiras nacionais, nunca foi tão relevante. O status único da OMS como uma organização baseada em ciência e evidências que define normas e padrões aplicáveis globalmente a torna vital em um mundo em rápida mudança. A atuação da Organização em prol da saúde e direitos humanos é indispensável para garantir que ninguém seja excluído. São necessários esforços amplos e contínuos para construir uma comunidade comprometida com o futuro compartilhado da humanidade, capacitando todas as pessoas a melhorar sua saúde, abordar os determinantes da saúde e responder aos desafios de saúde”(9).

Nessa perspectiva, uma atividade-chave a ser enfatizada no Plano de Trabalho deste CC é a capacitação em pesquisa em enfermagem, com foco no desenvolvimento de recursos humanos e educação. Por meio do estabelecimento de acordos acadêmicos com diferentes instituições da América Latina e do Caribe, este CC tem feito parcerias para a formação de profissionais de enfermagem, visando fortalecer a profissão nesses países e promover o aumento do conhecimento e da liderança de enfermeiras e enfermeiros em diferentes áreas de trabalho(10). Esta ação está alinhada ao ODS 17 e ao compromisso da OPAS em estabelecer parcerias com CCs para o desenvolvimento de capacitação, destacando os CCs “como um elemento-chave para a cooperação técnica fornecida não apenas pela Organização, mas também entre os países, como um exemplo bem-sucedido de pan-americanismo”(8). Desta maneira, por meio dos centros colaboradores, a OPAS também demonstra sua participação substantiva na consecução da meta 9 do ODS 17“Reforçar o apoio internacional para a implementação eficaz e orientada da capacitação em países em desenvolvimento, a fim de apoiar os planos nacionais para implementar todos os objetivos de desenvolvimento sustentável, inclusive por meio da cooperação Norte-Sul, Sul-Sul e triangular”(8). Isso enfatiza a natureza interdependente do progresso na formação de recursos humanos e a consequente melhoria no desenvolvimento do país. E é também uma forma de inclusão, respeitando as necessidades e peculiaridades de cada país. Segundo Buss, a Agência Brasileira de Cooperação “define a cooperação técnica internacional como um importante instrumento para o desenvolvimento, que apoia os países a promover mudanças estruturais sociais e econômicas, incluindo medidas estatais para o fortalecimento institucional. Os programas implementados em sua esfera de competência permitem a transferência ou compartilhamento de conhecimentos, experiências e melhores práticas, por meio do desenvolvimento de habilidades e capacidade institucional, a fim de promover um salto qualitativo duradouro”(11).

Outro ponto que também permeia a capacitação é a disseminação do conhecimento da enfermagem. Além das publicações de resultados de pesquisas em importantes periódicos de enfermagem, este CC desenvolve cursos de formação gratuitos para enfermeiras e enfermeiros da América Latina e Caribe. Por meio da qualificação profissional, espera-se que esses profissionais possam conquistar melhores empregos e posições profissionais e, consequentemente, uma possível retenção dessa força de trabalho em seus respectivos países. Isso também pode refletir na promoção da saúde e na melhoria do atendimento à população, promovendo, assim, não apenas a formação visando à prevenção e tratamento de doenças, mas também, o aumento da expectativa de vida saudável. Sabe-se que a expectativa de vida saudável envolve outras questões econômicas e sociais, que dependem de políticas públicas governamentais, no entanto, este CC fomenta iniciativas que podem ajudar a melhorar a qualidade de vida da comunidade. Além disso, como reforça Buss, a solidariedade global é essencial, priorizando particularmente as necessidades das populações mais pobres e vulneráveis(6).

Por fim, o fortalecimento de redes é essencial. Este CC reconhece que a construção de parcerias é fundamental para o desenvolvimento e melhoria da saúde global(12,13). A colaboração de profissionais e pesquisadores da América Latina, Caribe e África proporciona o desenvolvimento de todos os envolvidos e permite que soluções para os problemas de saúde sejam encontradas em conjunto. Isso também possibilita a disseminação do conhecimento nos países, permitindo o acesso a melhorias na saúde para sua população. Segundo o documento da OPAS/OMS, “uma rede regional e plataforma oficial para compartilhar e cruzar continuamente as experiências nacionais com as metas e implementação dos ODS promoverá muito a troca de conhecimento relacionado ao desenvolvimento sustentável e à equidade em saúde”(14). Em resumo, o fortalecimento das redes de enfermagem possibilita a organização de uma agenda que seja relevante para os problemas de saúde vivenciados local e regionalmente, a integração de diferentes profissionais de saúde e outras áreas relacionadas à saúde no desenvolvimento de pesquisas, e oportunidades de disseminação de conhecimento e colaboração com países da América Latina, Caribe e África e agentes multiplicadores.

Ainda há muitas dificuldades a serem superadas, principalmente em relação ao estabelecimento de políticas públicas governamentais que possam ser implementadas, tendo em vista benefícios para a população e financiamento fornecido por agências e universidades públicas para o desenvolvimento de pesquisas e disseminação do conhecimento. O idioma também se torna uma barreira para a disseminação do conhecimento desenvolvido em colaboração, mas esses desafios devem ser enfrentados em conjunto para que essas parcerias possam ser estabelecidas e as metas dos ODS alcançadas.

### Limitações do estudo

Os documentos são limitados temporalmente a 2018, pois ainda não havia resultados das atividades realizadas pelo CC quanto à seguinte designação, no momento da realização da pesquisa.

### Contribuições para o campo

Em síntese, o fortalecimento das redes de enfermagem permitirá trabalhar uma agenda pertinente aos problemas de saúde vivenciados local e regionalmente, integrar diferentes profissionais de saúde e outras áreas relacionadas à saúde no desenvolvimento de pesquisas, oportunidades de disseminação de conhecimento, colaboração com países da América Latina, Caribe e África e agentes multiplicadores.

## CONCLUSÕES

A análise das atividades realizadas pelo Centro Colaborador ao longo desses doze anos de designação conclui que ele cumpriu com sucesso sua missão de promover o desenvolvimento da pesquisa em enfermagem. Isso é evidente por meio de seus processos e produtos alinhados às metas dos ODS, incluindo produção de conhecimento, capacitação de recursos humanos para pesquisa, liderança em estratégias de disseminação de conhecimento e transferência de resultados de pesquisa para serviços de saúde, visando facilitar a transformação e fortalecer as redes de enfermagem.

## Data Availability

Os dados da pesquisa estão disponíveis no corpo do artigo.
